# Periodontal Phenotype Modification of Peri-Implant Soft Tissue Deficiency Using Subepithelial Connective Tissue Grafts and Bone Grafts in the Esthetic Region

**DOI:** 10.3390/medicina60060841

**Published:** 2024-05-21

**Authors:** Won-Bae Park, Roberto Gonzalez Yumar, Ji-Young Han, Philip Kang

**Affiliations:** 1 Department of Periodontology, School of Dentistry, Kyung Hee University, Seoul 02447, Republic of Korea; wbpdds@naver.com; 2 Private Practice in Periodontics and Implant Dentistry, Seoul 02771, Republic of Korea; 3 Division of Periodontics, Section of Oral, Diagnostic and Rehabilitation Sciences Columbia University College of Dental Medicine, #PH7E-110, 630 W. 168 St., New York, NY 10032, USA; rcg2146@cumc.columbia.edu; 4 Department of Periodontology, Division of Dentistry, College of Medicine, Hanyang University, 222-1 Wangsimni-ro, Seongdong-gu, Seoul 04763, Republic of Korea

**Keywords:** bone graft, dental implant, peri-implant mucosa, phenotype, subepithelial connective tissue graft

## Abstract

Peri-implant soft tissue deficiency (PSTD) is a significant factor impacting aesthetics, particularly in the anterior zone, where labial bone resorption and thin peri-implant phenotypes are common. The occurrence of a gray color around the implant fixture due to PSTD can be aesthetically concerning in the esthetic zone. In cases involving natural teeth, autogenous soft tissue grafts such as subepithelial connective tissue grafts (SCTGs), free gingival grafts (FGGs), and coronally advanced flaps (CAFs) are commonly utilized. However, there are limited reports of using bone grafts in conjunction with these techniques for modifying the gingival phenotype around both teeth and implants. In the presented cases where PSTD resulted in visible gray coloration of the implant fixture in the esthetic zone, mechanical and chemical decontamination of the exposed implant surface was performed using a titanium brush and tetracycline (Tc) HCl. Subsequently, to enhance peri-implant mucosa thickness and mask the titanium color, simultaneous SCTG and bone grafting procedures were conducted. Within the limitations of these case reports, successful esthetic outcomes were achieved and maintained without recurrence for 3–6 years following the simultaneous subepithelial connective tissue graft and bone graft procedures. These findings suggest the potential efficacy of this combined approach in addressing PSTD and enhancing aesthetic results around dental implants, though further studies are needed to validate these outcomes.

## 1. Introduction

Implant placement in the esthetic zone poses unique challenges for dental clinicians aiming to achieve optimal aesthetic outcomes. The implant platform must be positioned three-dimensionally to enable the fabrication of an ideal prosthesis that blends seamlessly with the surrounding dentition. Guided surgery using cone-beam computed tomography (CBCT) images can assist in achieving precise implant placement in these scenarios.

However, guided surgery can be particularly challenging in esthetic zones due to severe labial bone resorption and vertical bone loss [1]. Consequently, implants are often placed based on the available bone envelope, which may compromise the emergence profile of the prosthesis and overall aesthetics. Such compromises can lead to deficiencies in the peri-implant mucosa and affect the final aesthetic result. It is essential for clinicians to carefully assess bone anatomy and soft tissue contours in the esthetic zone during implant planning. Techniques such as bone augmentation or soft tissue grafting may be necessary to optimize the peri-implant mucosa and enhance the final aesthetic outcome of implant restorations in these challenging areas.

Deficiencies in the peri-implant mucosa, often characterized by a thin phenotype, can manifest within six months of implant prosthesis delivery in the esthetic area [2,3]. Contributing factors include buccal implant positioning, inappropriate implant diameter selection, a thin labial bone plate, and a thin biotype of peri-implant mucosa [2,3]. Addressing these challenges in the esthetic zone requires a comprehensive approach. Socket preservation or augmentation is necessary to prepare the implant site within the extraction socket, followed by labial bone augmentation once healing is complete. Traditional techniques using free gingival grafts (FGGs), subepithelial connective tissue grafts (SCTGs), and coronally advanced flaps (CAFs) have been conventionally used to modify gingival phenotypes around natural teeth [4].

However, managing peri-implant soft tissue deficiencies (PSTDs) lacks well-established treatment protocols and long-term outcomes compared to natural teeth. As a result, clinicians often adapt techniques traditionally used for teeth to address peri-implant soft tissue deficiencies, emphasizing the need for further research and refined approaches tailored specifically for implant sites.

In the management of denuded root surfaces and peri-implant soft tissue deficiencies (PSTDs), the subepithelial connective tissue graft (SCTG) combined with a coronally advanced flap (CAF) is considered a gold standard technique based on the existing literature [5,6]. Additionally, alternative approaches such as guided bone regeneration (GBR), tunnel techniques, and partly epithelized connective tissue grafts have been employed [7,8,9,10]. In cases where autogenous soft tissue grafting may not be feasible or preferred, acellular dermal matrix and collagen matrix substitutes have emerged as viable alternatives [11]. However, there remains variability in the selection of surgical methods and outcomes, influenced by factors such as Miller’s classification of gingival recession, the specific tooth or implant arrangement, the number of sites to be treated, the availability of donor tissue, and the expertise of the treating clinician [4]. This heterogeneity underscores the importance of individualized treatment planning and surgical decision-making based on patient-specific factors and clinician experience to achieve optimal outcomes in addressing denuded roots and peri-implant soft tissue deficiencies.

The cases presented here share common characteristics of a thin peri-implant soft tissue phenotype and exposure of the titanium implant surface due to labial bone resorption. In addressing these challenges, both bone grafting and subepithelial connective tissue grafts (SCTGs) were deemed necessary. While there are reports of simultaneous use of SCTGs and bone grafting for modifying gingival phenotypes and achieving root coverage around natural teeth [12], to our knowledge, there have been no reports of similar cases involving peri-implant soft tissue deficiencies (PSTDs) with concurrent bone grafting.

This report introduces a predictable surgical technique aimed at addressing the gray shade of an exposed implant surface resulting from labial bone resorption in the esthetic zone with a thin soft tissue phenotype. The technique utilizes both SCTG and bone grafting to enhance soft tissue volume and mask the gray appearance associated with titanium implant exposure.

## 2. Case Presentation and Results

This report encompasses four distinct cases, all of which exhibited PSTD on implants previously placed within the esthetic zone. Both SCTG and bone grafting were employed to address PSTD. The demographic details of the patients are outlined in Table 1.

### 2.1. Case 1

**Figure 1:**
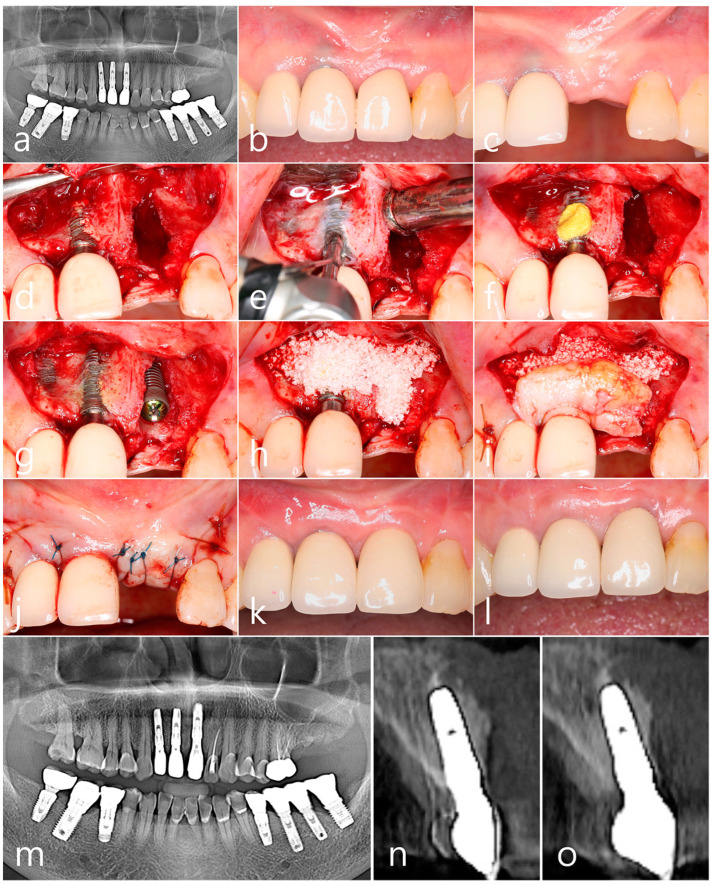
Case 1. (**a**) In panoramic radiography, the interproximal bone level of implant #11 was well maintained, but space widening was observed in implant #21 with loss of osseointegration; (**b**) The gray color of the fixture is seen through the peri-implant mucosa according to the resorption of the #11 implant’s labial bone; (**c**) Implant #21 was removed, and implant placement was planned 2 months later; (**d**) After the labial mucoperiosteal flap was reflected, resorption of the facial bone and exposure of the implant surface of implant #11 was observed; (**e**) A rotary titanium brush was used to decontaminate the exposed surfaces of implants #11 and #12; (**f**) Additional chemical detoxification was performed with Tc HCl for 5 min. The granulation tissue in the extraction socket of the #21 implant was thoroughly debrided; (**g**) After sufficient saline irrigation, a new implant was placed 3.0 mm subcrestally from the adjacent proximal bone at site #21; (**h**) Labial bone augmentation was performed using a synthetic bone graft substitute; (**i**) An SCTG was acquired by a trap-door approach in the left palate and fixed with a 4-0 catgut through a sling suture in implant #11; (**j**) The overlying mucoperiosteal flap was closed; (**k**) Uncovering was performed 6 months after surgery, and the final prosthesis was delivered 2 months later. Esthetics in the #11 implant were restored; (**l**) In the clinical findings 4 years after surgery, the esthetics of implant #11 were well maintained, and the gingival level was not changed. Implant #21 showed a slight gingival recession. (**m**) No change in the marginal bone level was observed in panoramic radiography taken 4 years later; (**n**) A cross-sectional image of CBCT taken after prosthesis was delivered; (**o**) Some resorption of labially augmented bone was observed in cross-sectional images of CBCT taken 4 years after prosthesis was delivered.

A 70-year-old female non-smoker patient had implants #11, #12, and #21 placed using a flapless technique three years prior. Panoramic radiography showed that while the interproximal bone level of implant #11 was well maintained, there was a noticeable space widening with loss of osseointegration in implant #21 (Figure 1a). In the clinical findings, 3 years after prosthesis delivery, a gray titanium color was seen according to the resorption of the #11 implant’s facial bone. This is PSTD Class I, according to Zucchelli et al. [3]. Implant #21 developed mobility due to a loss of osseointegration (Figure 1b). Implant #21 was removed, and implant placement was planned 2 months later. The extraction socket of implant #21 healed well (Figure 1c). Under local anesthesia, vertical incisions were made at the distobuccal line angle of the #11 implant and the mesiobuccal line angle of the #21 tooth. After the midcrestal incision, the labial mucoperiosteal flap was reflected. Resorption of the facial bone and exposure of the implant surface of implant #11 were observed (Figure 1d). A rotary titanium brush (Dentium, Suwon, Republic of Korea) was used to decontaminate the exposed areas of implants #11 and #12 (Figure 1e). Additional chemical detoxification was performed with tetracycline (Tc) HCl (Chong Kun Dang Pharmaceutical., Seoul, Republic of Korea) for 5 min. The granulation tissue in the extraction socket of the #21 implant was thoroughly debrided (Figure 1f). After sufficient saline irrigation, a new implant was placed 3.0 mm subcrestally from the adjacent proximal bone at site #21 (Figure 1g). Lateral bone augmentation was performed using a synthetic bone graft substitute (Osteon III, Genoss, Suwon, Republic of Korea) (Figure 1h). An SCTG was acquired by a trap-door approach in the left palate and fixed with a 4-0 catgut through a sling suture in the #11 implant. The SCTG was placed in a more coronal position (Figure 1i). The overlying mucoperiosteal flap was closed with 4-0 nylon (Figure 1j). No events were found in the postoperative healing process. Uncovering was performed 6 months after surgery, and the final prosthesis was delivered 2 months later. In implant #11, the esthetics were restored (Figure 1k). In the clinical findings 4 years after surgery, the esthetics of the #11 implant were well maintained, and 1 mm gingival recession was observed in the #21 implant (Figure 1l). In panoramic radiography taken 4 years after prosthesis delivery, there was no change in the crestal bone level around the implant (Figure 1m). Labially augmented bone was observed on the buccal side of the implant in cross-sectional images of CBCT taken after the prosthesis was delivered (Figure 1n). Less than 2 mm of labial bone resorption was observed in cross-sectional images of CBCT taken 4 years after the prosthesis was delivered (Figure 1o).

### 2.2. Case 2

**Figure 2:**
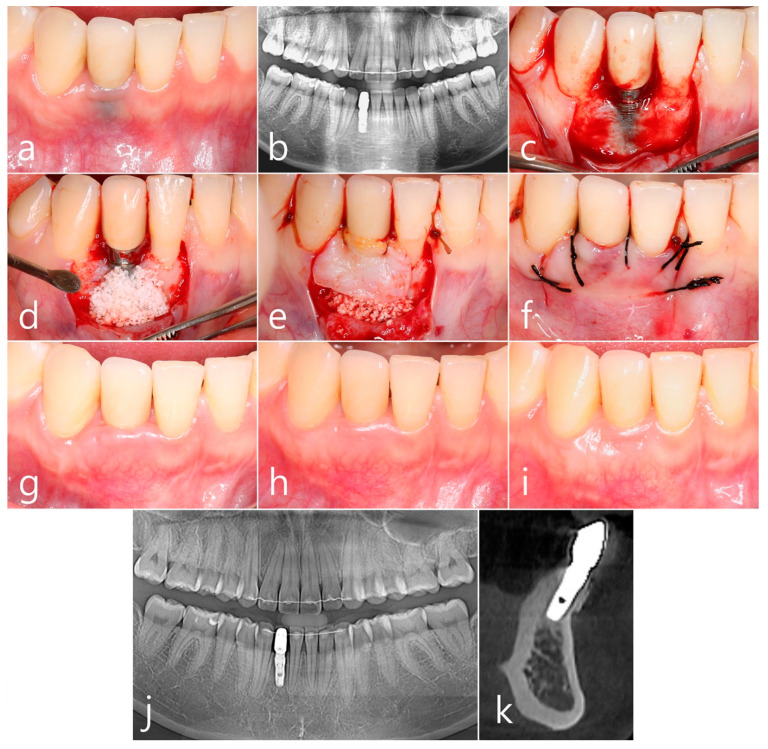
Case 2. (**a**) 3 years after prosthesis delivery, the gray color of implant #42 was shown through the peri-implant mucosa. The phenotype of the labial peri-implant mucosa was also very thin; (**b**) No resorption of interproximal bone was observed in panoramic radiography; (**c**) Vertical incision and crevicular incision were performed on the line angle of the adjacent teeth of the #42 implant, and the mucoperiosteal flap containing the interdental papilla was reflected. The labial surface of the implant was exposed. It was decontaminated with a rotary titanium brush. In addition, chemical detoxification was performed using Tc HCl; (**d**) A synthetic bone graft substitute was grafted around the exposed titanium surface; (**e**) An SCTG obtained from the right palatal surface was closed with a 4-0 catgut; (**f**) The overly mucoperiosteal flap was closed with 4-0 black silk; (**g**–**i**) According to clinical findings 6 months after the procedure, 3 years after the procedure, and 7 years after the procedure, the gingival phenotype was improved, and the titanium color was well blocked, so the esthetics were restored; (**j**) No loss of crestal bone was observed in panoramic radiography taken 7 years after the procedure; (**k**) In the CBCT image taken 7 years after the procedure, the labially augmented bone of the implant was well consolidated and corticalized, and sufficient thickness was maintained.

A 35-year-old female non-smoker patient received a Ø 3.8 × 10 mm implant (Implantium, Dentium, Suwon, Republic of Korea) placed at the missing site of tooth #42 after orthodontic treatment. There was no exposure of the implant at the time of surgery. The prosthesis was placed 4 months later, and no esthetic deficit occurred. After 3 years of prosthesis delivery, the patient returned to the clinic because of a change in gingival color. The gray color of implant #42 was shown through the peri-implant mucosa. The phenotype of the labial peri-implant mucosa was also very thin (Figure 2a). No interproximal bone resorption was observed in panoramic radiography (Figure 2b). This is PSTD Class I, according to Zucchelli et al. [3]. Under local anesthesia, vertical incisions and crevicular incisions were performed on the line angle of the adjacent teeth of implant #42, and mucoperiosteal flaps containing the interdental papilla were reflected. Part of the implant surface was exposed due to the resorption of the implant’s facial bone plate. It was decontaminated with a rotary titanium brush (Dentium, Suwon, Republic of Korea). In addition, chemical detoxification was performed using Tc HCl (Figure 2c). A synthetic bone graft substitute (Osteon II, Genoss, Suwon, Republic of Korea) was grafted around the exposed titanium surface (Figure 2d). An SCTG obtained from the right palatal surface was closed with a 4-0 catgut (Figure 2e). The overlying mucoperiosteal flap was closed with 4-0 black silk (Figure 2f). According to the clinical findings, 6 months, 3 years, and 7 years after the procedure, the gingival phenotype was improved, the titanium color was well covered, and the aesthetics were restored (Figure 2g–i). No loss of crestal bone was observed in panoramic radiography taken 7 years after the procedure (Figure 2k). In the cross-sectional image of CBCT taken 7 years after prosthesis delivery, well-augmented bone was observed, and consolidation was well achieved.

### 2.3. Case 3

**Figure 3:**
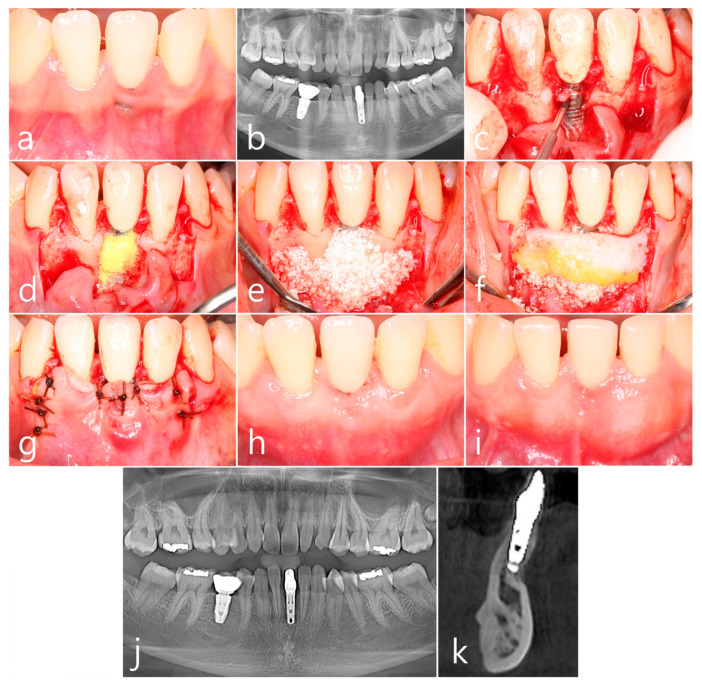
Case 3. (**a**) Peri-implant soft tissue dehiscence occurred in the labial gingiva 3 years after implant #31 was placed; (**b**) No crestal bone loss was found in panoramic radiography; (**c**) A vertical incision was performed in the distal line angle of the adjacent tooth of implant #31, and a crevicular incision was performed to include the buccal interdental papilla. Next, the labial mucoperiosteal flap was reflected. The implant surface was exposed on the labial surface of implant #31. Surface decontamination was performed using a rotary titanium brush; (**d**) Additionally, chemical detoxification was performed with Tc HCl. Sufficient saline irrigation was performed; (**e**) A synthetic bone graft substitute was used for bone grafting; (**f**) An SCTG obtained from the left palatal surface was fixed with 4-0 catgut on the bone graft; (**g**) A mucoperiosteal flap was also closed with a 4-0 catgut; (**h**) In the clinical finding 1 month after the procedure, the dehiscence of the peri-implant mucosa disappeared, and the esthetics were restored; (**i**) In the clinical findings 4 years after the procedure, the thickness of the peri-implant mucosa was further increased; (**j**) No change in crestal bone was observed in panoramic radiography taken 4 years after the procedure; (**k**) Labially augmented and corticalized bone of implant #31 was observed in cross-sectional images of CBCT taken 4 years after surgery.

A 25-year-old female non-smoker patient developed peri-implant dehiscence in the buccal gingiva 3 years after implant #31 was placed. The placed implant was a Ø 3.3 × 10 mm Zimmer TSV implant (Zimmer Biomet, Warsaw, IN, USA) (Figure 3a). No crestal bone loss was found in the panoramic radiography (Figure 3b). This is PSTD Class II, according to Zucchelli et al. [3]. Under local anesthesia, a vertical incision was made in the distal line angle of the adjacent teeth of the #31 implant, and a crevicular incision was made to include the buccal interdental papilla. The buccal mucoperiosteal flap was reflected. The implant surface was exposed on the buccal surface of implant #31. Surface decontamination was performed using a rotary titanium brush (Dentium, Suwon, Republic of Korea) (Figure 3c). Additionally, chemical detoxification was performed with Tc HCl. Sufficient cleaning was achieved by saline irrigation (Figure 3d). A bone graft was performed using a synthetic bone graft substitute (Osteon III, Genoss, Suwon, Republic of Korea) (Figure 3e). An SCTG obtained from the left palatine surface was fixed to the bone graft with 4-0 catgut (Figure 3f). The overlying mucoperiosteal flap was also closed with a 4-0 catgut (Figure 3g). In the clinical picture, 1 month after the procedure, the dehiscence of the peri-implant mucosa disappeared, and the esthetics were restored (Figure 3h). In the clinical findings, 4 years after the procedure, the thickness of the peri-implant mucosa increased (Figure 3i). No changes in the crestal bone were observed in panoramic radiography taken 4 years after the procedure (Figure 3j). Augmented bone was observed on the labial surface of implant #31 in a cross-sectional image of CBCT taken 4 years after the procedure (Figure 3k).

### 2.4. Case 4

**Figure 4:**
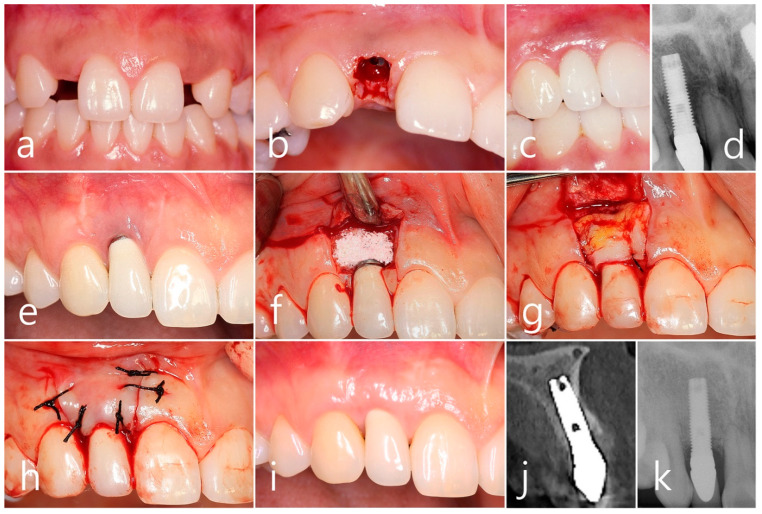
Case 4. (**a**) An external hexed implant of Ø 4.0 × 13 mm # 12 was placed using the flapless technique; (**b**) Uncovering was performed 4 months after the cover screw was inserted; (**c**) After 2 months, the final prosthesis was delivered; (**d**) An intraoral radiography was taken; (**e**) After 15 years, the patient underwent surgical intervention for the treatment of peri-implantitis. The metal collar of the prosthesis was exposed by gingival recession; (**f**) A mucoperiosteal flap was reflected through two vertical incisions and a horizontal incision at the cemento-enamel junction. A synthetic bone graft substitute was filled after surface treatment with a titanium brush and Tc HCl; (**g**) The SCTG was fixed with 4-0 catgut; (**h**) The mucoperiosteal flap was closed with 4-0 black silk; (**i**) In clinical findings 5 years after surgical intervention to resolve PSTD, the gingival recession was covered; (**j**) A well-preserved bone tissue was observed on the labial side of implant #12 in a cross-sectional image of CBCT taken 5 years later; (**k**) Slight interproximal bone loss was observed on the intraoral radiography taken 5 years later.

A 28-year-old female non-smoker patient sought treatment at the clinic to place an implant in the missing area of the maxillary lateral incisor (Figure 4a). Under local anesthesia, a Ø 4.0 × 13 mm hydroxyapatite-coated external hexed implant (Lifecore Biomedical, USA) was placed using the flapless technique. Uncovering was performed 4 months after the cover screw was inserted (Figure 4b). After 2 months, the final prosthesis was delivered (Figure 4c), and an intraoral X-ray was taken (Figure 4d). After 15 years, the patient returned to the clinic with PSTD, including gingival recession. The patient underwent surgical intervention for peri-implantitis at another clinic. This is PSTD Class II, according to Zucchelli et al. [3]. An additional surgical intervention was planned to treat PSTD (Figure 4e). Under local anesthesia, the mucoperiosteal flap was reflected through two vertical incisions and a horizontal incision at the cemento-enamel junction. A synthetic bone graft substitute (Osteon II, Genoss, Suwon, Republic of Korea) was filled after mechanical and chemical decontamination was performed on the exposed implant (Figure 4f). The SCTG was immobilized (Figure 4g), and the mucoperiosteal flap was sutured with 4-0 black silk (Figure 4h). In clinical findings, 5 years after surgical intervention to resolve PSTD, the gingival recession was covered (Figure 4i). In the cross-sectional image of CBCT taken 5 years later, well-preserved bone tissue was observed on the buccal side of implant #12 (Figure 4j). A slight crestal bone resorption was observed on an intraoral X-ray taken 5 years later (Figure 4k).

## 3. Discussion

To the best of our knowledge, there have been no prior case reports documenting the simultaneous use of subepithelial connective tissue grafts (SCTGs) and bone grafts for the surgical treatment of peri-implant soft tissue deficiency (PSTD).

Across the four cases presented, initial implants were placed and restored without notable esthetic concerns. However, during subsequent follow-up visits, various findings, including gingival recession, inflammation, thread exposure, and a visible gray hue, were observed. These esthetic issues were addressed through implant surface detoxification, followed by additional augmentations. Cases #1 and #2 were classified as PSTD Class I, while cases #3 and #4 were categorized as PSTD Class II, according to Zucchelli et al.’s classification [3]. However, it is noteworthy that each case underwent simultaneous SCTG and bone grafting, deviating from the recommended surgical approach outlined by Zucchelli et al. [3]. Over a follow-up span of 4 to 7 years post-surgery, there were no detectable clinical or radiological complications associated with the bone grafts. There was no penetration of bone graft particles into the soft tissue, nor was there any resultant infection. Improvements were noted in tissue thickness, aesthetics, and resolution of preoperative PSTD. Furthermore, CBCT imaging revealed that the interaction between the vascular bone surface and the bone graft led to graft consolidation and corticalization.

The primary goal of bone grafting in this context is to augment the thickness of the facial bone adjacent to the exposed implant surface. While direct bone formation on the avascular implant surface is not expected, the bone graft serves a critical role beyond simple biological filling. It effectively addresses the gray hue of the implant by promoting tissue thickening and aiding in bone maturation over time. This combined approach, incorporating both SCTG and bone grafting, offers a more comprehensive solution for managing PSTD compared to traditional methods reliant solely on SCTGs and coronally advanced flaps (CAFs). By addressing both soft tissue and underlying bone deficiencies simultaneously, this technique aims to optimize aesthetic outcomes and long-term stability around dental implants.

It was reported that the thickness of the labial bone plate and the gingival thickness were correlated with each other [13]. It is generally recommended that the facial bone thickness around dental implants be at least 1.5 mm to support long-term implant success and soft tissue stability [14]. This is because an apparatus such as the periodontal ligament of a tooth does not exist in an implant. When the implant is placed in the esthetic zone, it is necessary to secure the maximum buccal bone width by a more palatal/lingual position, a narrow implant diameter, and subcrestal placement of the implant platform [15]. However, this may be difficult to achieve, depending on the condition of the patients. After tooth loss, the buccal bone is rapidly resorbed over time. In the esthetic zone, the esthetics of the implants are often compromised by resorption of the facial bone plate or exposure to the gray titanium color.

Surgical procedures to cover localized gingival recession were reported to include a CAF, a laterally positioned flap, and a semilunar flap in addition to the SCTG [5]. In multiple teeth, simultaneously covering the denuded root and increasing the width of the keratinized mucosa requires the selection of an effective procedure and the skill of the operator. Even with implants, the first procedure considered to change the soft tissue phenotype and treat PSTD is an SCTG and a CAF [16,17,18].

There are very few studies on surgical methods of treating PSTD, unlike teeth, and long-term reliable data are lacking. The key to PSTD is the treatment method of the exposed implant surface and the need for a bone graft. Root coverage of the denuded root surface or modification of the periodontal soft tissue phenotype requires root planing and chemical detoxification procedures, and the evidence is sufficient [19]. However, the practice of surface treatment of exposed implants lacks evidence, and successful detoxification is difficult to achieve. Most of the decontamination methods for exposed implant surfaces studied so far were applied to peri-implantitis or exposure to the maxillary sinus [20]. Exposure of the facial peri-implant surface due to PSTD is fundamentally different from implant exposure due to peri-implantitis. Implant exposure due to PSTD is due to the resorption of facial bone and is not caused by infection but by insufficient securing of facial bone, so a better result than peri-implantitis is expected. In general, implant surface decontamination methods include titanium curettes, titanium brushes, and air-powder abrasives [21,22], and chemical decontamination methods include chlorhexidine, citric acid, and Tc HCl [23]. Recently, the use of a laser has been reported [24]. However, since no one method has a superior effect, a combination of several methods is recommended [25]. In these cases, a titanium brush and Tc HCl were used together.

Furthermore, there has been no report on the operation of additional bone grafts on SCTG. Bone graft substitutes must be grafted to the vascular surface to achieve consolidation and corticalization of the bone graft, but the advantages and role of grafting to an avascular surface such as the implant surface are unknown. However, Park et al. reported that satisfactory clinical and radiographic outcomes were achieved by using a combination of SCTG and bone grafts for root coverage of teeth and modification of periodontal phenotype [12]. In PSTD, the primary goal is achieved because the labial tissue thickness of the peri-implant mucosa is increased even if the bone graft is performed on the exposed implant surface, and the facial bone of the adjacent tooth acts as a biologic filler rather than a foreign body. Corticalization and consolidation of labially augmented bone were confirmed in CBCT images. Therefore, bone grafts have clinical significance beyond the resolution of PSTD.

In the present procedure, a barrier membrane was not used for GBR after a bone graft. The question is whether connective tissue can act as a barrier membrane. If it cannot play the role of a barrier membrane, it would be adequate to use a barrier membrane during surgery. Currently, there are reports that SCTG can also act as a barrier membrane [26,27,28]. In the future, the role of SCTG as a barrier membrane and the nature of contact with the bone graft-exposed implant surface (for example, biologic inert adaptation or osseointegration) should be confirmed through surgical re-entry and histological examination.

This case report has the disadvantage of lacking critical factors such as the limitation of a case number, a lack of data on the dimensions of the ridge during the first implant placement, surgical re-entry, and histologic examination. It is hoped that future research will be conducted to secure evidence.

## 4. Conclusions

In the context of the current case series, observed over a follow-up period of 4 to 7 years post-surgery, the combined application of a subepithelial connective tissue graft (SCTG) and a bone graft showcased effectiveness in addressing peri-implant soft tissue deficiencies (PSTDs).

## Figures and Tables

**Table 1:** Patients’ demographic information.

Case	Age/Sex	Smoking	Implant Site	Implant Site (mm)	Follow-Up Period (Years)
1	70/F	No	#21	3.8 × 12	4
2	35/F	No	#42	3.8 × 10	7
3	25/F	No	#31	3.3 × 10	4
4	28/F	No	#12	3.8 × 13	5

## Data Availability

The data that support the findings of this study are available from the corresponding author upon reasonable request.
